# “Waking up” the sleeping metaphor of normality in connection to intersex or DSD: a scoping review of medical literature

**DOI:** 10.1007/s40656-022-00533-8

**Published:** 2022-10-25

**Authors:** Eva De Clercq, Georg Starke, Michael Rost

**Affiliations:** 1 Institute for Biomedical Ethics, University of Basel, Bernoullistrasse 28, 4056 Basel, Switzerland; 2 Institute of Biomedical Ethics and History of Medicine, University of Zürich, Winterthurerstrasse 30, 8006 Zurich, Switzerland; 3 College of Humanities, École Polytechnique Fédérale de Lausanne, Rte Cantonale, 1015 Lausanne, Switzerland

**Keywords:** DSD, Ethics, Intersex, Normality, Phenotype

## Abstract

The aim of the study is to encourage a critical debate on the use of normality in the medical literature on DSD or intersex. For this purpose, a scoping review was conducted to identify and map the various ways in which “normal” is used in the medical literature on DSD between 2016 and 2020. We identified 75 studies, many of which were case studies highlighting rare cases of DSD, others, mainly retrospective observational studies, focused on improving diagnosis or treatment. The most common use of the adjective normal was in association with phenotypic sex. Overall, appearance was the most commonly cited criteria to evaluate the normality of sex organs. More than 1/3 of the studies included also medical photographs of sex organs. This persistent use of normality in reference to phenotypic sex is worrisome given the long-term medicalization of intersex bodies in the name of a “normal” appearance or leading a “normal” life. Healthcare professionals should be more careful about the ethical implications of using photographs in publications given that many intersex persons describe their experience with medical photography as dehumanizing.

## Introduction

### Language matters, language wounds

Thirty years ago, the medical anthropologist Emily Martin published a thought-provoking article on gender-biased language in scientific accounts of reproductive biology: “The Egg and the Sperm: How Science has constructed a Romance based on Stereotypical Male–Female Roles” (Martin, 1991). Her main message was that scientists have instilled the egg and the sperm with cultural stereotypes that portray women as passive and men as active. She urged us “to wake up sleeping metaphors in science” to become aware of their social effects and “to rob them of their power to naturalize social conventions” (Martin, 1991, p. 501).

Although in humanistic disciplines the focus on medical discourse is far from new (Foucault, 1978, 1980), within medical literature, discussions on medicine and language have largely focused on considerations regarding clinical communication and doctor-patient interactions (Franz & Murphy, 2018). The field of narrative-based medicine—founded by Rita Charon at Columbia University (Charon, 2006)—with its emphasis on illness stories as a way to improve patient welfare, is exemplary here.

But why is it important to extend communicative competence in clinical practice beyond communication skills? Why should we consider to include also a critical reflection on word choice? Scholars working within critical race theory (Matsuda, 1993) and gender studies (Butler, 1997), have shown that words can cause injury like sticks and stones. These researchers have taken their cue from John Austin’s *How to do things with words* (Austin, 1962). In this seminal work, the philosopher of language drew attention to the performative quality of language, i.e. words do not merely describe or report, but actually bring about things in the world. Hate speech, for example, is said to be inherently harmful and to have detrimental consequences insofar as it undermines the target group’s assurance of inclusion in society and may incite and condone acts of violence and discrimination against them (Waldron, 2012).

Although medical language is generally presumed to be a neutral tool, used to describe a patient’s medical condition and the required treatment, recent research highlights how stigmatizing language in patient records shapes physician perspectives of a person (Goddu et al., 2018). The study of Goddu and colleagues shows that clinicians who read these records—without knowing the patient—often take on a negative attitude towards the patient and provide less strong pain management (Goddu et al., 2018). Hence, biased language shapes the way healthcare professionals think and act, and these thoughts and actions in turn affect quality of care and risk contributing to persistent health disparities. This might explain why Marcus and Snowden in their recent commentary on sexual health (Marcus & Snowden, 2020) are concerned about the persistent use of ambiguous and stigmatizing language such as “unsafe sex,” or “risky sex,” in the scientific literature on sexual behavior. Their concern is that these risk-based messages, aside from downplaying the importance of a positive approach to sexuality, contribute to HIV and STI (sexually transmitted infections) stigma which may prevent people from seeking sexual health care and negatively influence healthcare professionals who provide sexual healthcare.

As a visual language, photography should be exposed to the same scrutiny as written (and verbal) language. In fact, far from being a mere tool used to capture an objective reality, photography also offers an interpretation of the world. This is testified by the phenomenon of embedded reporting in war situations (Butler, 2009). This means that photography is a tool of power, or as Susan Sontag (Sontag, 1978, p. 175) states: “To photograph is to appropriate the thing photographed. It means putting oneself into a certain relation to the world that feels like knowledge and, therefore, like power”. Medical photography too—although a seemingly objective means for clinical documentation, education and publication purposes—is not without risks for patient’s privacy and autonomy and thus may necessitate informed consent (Kazemi et al., 2019). Moreover, according to social scientist Sarah Topp, medical photography is situated in a culture of surveillance that risks to reduce human experience to diagnostic categories (Topp, 2015). Still, despite certain legal limits concerning predominantly privacy rights, no direct guidance exists on how to deal with the ethical challenges regarding the dissemination, ownership and storage of medical photographs (Harting et al., 2015).

### Language and the care of persons born with variations of sex characteristics

The debate around terminology has been particularly salient in the health care management of intersex persons or individuals born with variations in sex characteristics (Feder, 2009; Lundberg et al., 2018). For healthcare professionals the language used to refer to these variations should be descriptive, accurate and transparent to enable adequate medical care (Hughes, 2015; Pasterski et al., 2010). Researchers in the humanities are instead concerned about the socio-cultural norms that are hidden in these apparently neutral terms that risk labelling intersex persons as defective and in need of correction (Davis, 2015; Topp, 2013).

Since the 2006 *Chicago Consensus Statement* (Lee et al., 2006), organized by the Lawson Wilkins Pediatric Endocrine Society and the European Society for Pediatric Endocrinology, the diagnostic nomenclature for these variations is “disorders of sex development” or DSD, defined as congenital conditions in which the development of chromosomal, gonadal, or anatomic sex is considered atypical (Lee et al., 2006). The acronym DSD has been largely adopted by the medical profession. It has replaced older terms (e.g. hermaphrodite, intersex, sex reversal) which were considered sensationalizing, stigmatizing and confusing for patients and scientifically meaningless for clinicians (Hughes, 2015; Pasterski et al., 2010). The revised terminology has gone hand in hand with a new classification system that moves away from associations with gender and is grounded in genetics, in particular in karyotype analysis. A karyotype is the visual depiction of all the chromosomes in a cell which generally contains 23 pairs of chromosomes of which 22 pairs are called autosomes and one pair are the so-called sex chromosomes (e.g. XX for genotypic female and XY for genotypic male). The three main diagnostic categories within the classification are: sex chromosome DSD, 46 XY DSD and 46 XX DSD.

The new nomenclature and the DSD classification system are not beyond dispute. They have been heavily criticized by patient advocates (Delimata et al., 2018; Feder, 2009; Reis, 2009), affected persons, support groups and scholars in the humanities (Griffiths, 2018). For them, the first part of the acronym, “disorder”, risks to turn bodily variations into pathological conditions necessitating medical interventions whereas most variations do not cause urgent health problems (Amato, 2016; Holmes, 2008; Topp, 2013). Moreover, critics of DSD often point out that such variations are much more common than most people think and thus far from “abnormal” (Rich et al., 2016).

The concern with over-medicalization is not surprising. For most of the twentieth century, under influence of the optimal gender policy of the sexologist John Money (Money et al., 1955), irreversible hormonal and surgical treatments were performed to “normalize” infants born with variations in sex characteristics, often with dramatic consequences, such as loss of sexual sensation or incontinence (Creighton et al., 2002). Furthermore, for decades, repeated medical examinations and medical photography of genitals were part of the standard of care for intersex children (Creighton et al., 2002). For many persons with variations of sex characteristics, this persistent exposure to the medical gaze has led to long-term psychological distress (Creighton et al., 2002; Topp, 2013).

Some of the major outcomes of the *Chicago Consensus Statement* (Lee et al., 2006) are (1) the recommendation to postpone medically unnecessary interventions until the child is competent to decide, (2) the emphasis on open and ongoing communication with families and (3) the need for holistic, multidisciplinary care (i.e. comprising not only medical care, but also psycho-social support). Still, for many advocacy groups the consensus statement has failed to protect intersex people from harmful practices in the name of a “normal” appearance and a “normal” life (Feder, 2009; Lundberg et al., 2018): despite increased skepticism, cosmetic operations have not been ruled out by the *Consensus* as they might reduce parental distress and improve bonding (Feder, 2009). Hence, a general moratorium on sex assignment surgeries does not exist (Gardner & Sandberg, 2018; Hughes, 2015; Sandberg et al., 2017; Wiesemann et al., 2010). The Consensus has thus been criticized for privileging a biomedical narrative over and against a focus on persons’ lived experience (Griffiths, 2018) and for justifying the practice of unnecessary medical interventions. In most countries, such surgeries continue to be performed (Monro et al., 2017) despite the (soft law) resolutions that have been issued against them by the Council of Europe (Council of Europe, 2015) and the World Health Organization (World Health Organisation, 2015). This does not mean that all medical treatment is harmful, on the contrary. People with VSC need specialized intersex health care, i.e. care that takes into account the individual differences among people with VSC (health risks, experiences, functional capabilities) and includes not only clinical care but also psycho-social care, sexual health care and fertility counselling.

Although many activists and affected persons are critical about the label DSD, and might reclaim intersex as an identity to be proud of, not all people concerned identify as intersex. Some might prefer other terms such as variation, difference, divergence, or the name of their syndrome or even use different terms depending on the situation (Lundberg et al., 2018). This shows that experts by experience have strong differing opinions on which terms to use to make sense of their embodiment and, consequently, demand linguistic flexibility; a flexibility which seems incongruent with the medical call for a clear and transparent umbrella term (Lundberg et al., 2018). Hence, “health professionals need to take seriously the power invested in biomedical knowledge and the effects that such knowledge might have on people’s bodies, lives and identities” (Lundberg et al., 2018, p. 164).

### Aims

The concept of normality is prominent in the field of medicine insofar diagnosis, treatment, and health, are based upon the delineation between a normal and abnormal functioning body (Chadwick, 2017). This is testified by the frequent use of the term “normal” in the International Classification of Diseases (ICD-11) (Rost, 2021) Nevertheless, there is currently no clear-cut definition of normality in medical literature and it seems to be used both in a descriptive and normative or evaluative way (Catita et al., 2020; Chadwick, 2017). The latter approach towards normality is worrisome because it might contribute to the medical desire to “mould individuals to the perceived norm rather than embracing difference” (Chadwick, 2017, p. 11).

The latter concern is of particular relevance for persons born with intersex variations given the long-standing history of normalizing cosmetic surgery. DSD is included or referred to in four different chapters of ICD-11, namely in chapter 5 (endocrine, nutritional and metabolic diseases), chapter 16 (diseases of the genitourinary system); chapter 17 (conditions related to sexual health); and chapter 20 (developmental anomalies). The nomenclature used to refer to variations of sex characteristics are DSD and hermaphroditism. The term intersex is never used. Other words used in reference to DSD are: mal-formative disorders of sex development, sex chromosomes anomaly, abnormal genital development, pathological, and defect. These diagnostic categories inform clinical practice and treatment of intersex bodies, and risk to overlook the difference between individual bodily variation and bodily processes in need of medical intervention (Lundberg et al., 2018).

The aim of the present paper is to “wake up” the sleeping metaphor of normality in connection to DSD in the medical literature. In particular, the study aims to identify peer-reviewed publications in medical journals to (1) map the various ways in which normal/normality is used in relation to DSD, (2) explore whether the use of normal/normality somehow informs clinical care and (3) identify any critical discussion on the use of “normal/normality”. The study will inform mainly healthcare professionals working in the field of DSD.

## Methods

A scoping review was conducted to identify and map the various ways in which “normal” is used in the medical literature on DSD or intersex. Given this broad scope, the data come from different types of study design (observational studies, experimental studies, qualitative, quantitative, theoretical studies, and interventions). In line with other scoping reviews, no quality assessment or critical appraisal of the included studies was conducted (Munn et al., 2018; Peters et al., 2015). The following online databases were systematically searched: Scopus, PubMed, PsycInfo, CINAHL, & Embase to cover the medical, nursing, and mental health research on the topic (see Table 1). The research terms were identified based on background reading on intersex and normality and on intra-team discussions. To capture all relevant results, the search terms were combined using Boolean logic (see Table 1). Inclusion criteria were: (1) published in peer-reviewed medical journals; (2) published between 2016 (publication of the updated version of Chicago convention on the clinical management of DSD) and July 2020; (3) focus on medical management (i.e. classification, diagnosis, treatment) of DSD; (4) inclusion of the term (ab)normality, (ab)normality in the abstract or full text, (5) written in English, German or French. No restriction was placed on the type of methodology. Conference abstracts, letters, dissertations, and systematic or scoping literature reviews were excluded. Given that the current classification system of DSD is not beyond discussion (and disagreements about how to define intersex/DSD continue to persist) all papers that refer to DSD or intersex were included.

**Table 1:** Search terms

Matches^1^						
No	Search terms	Scopus	PubMed	PsycINFO	CINAHL	Embase
1.	normal* OR abnormal* OR anormal* OR subnormal*	766.785	404.576	37.043	101.305	668.366
2.	intersex* OR “disorder of sexual development” OR “difference of sexual development “ OR “variation of sex characteristics” OR “disorder of sexual differentiation”	1.597	709	292	289	688
3.	1) AND 2)	223	95	12	51	131
Total results: 512						

Date of last search: 30.07.2020; ^1^2016–2020

**Fig. 1:**
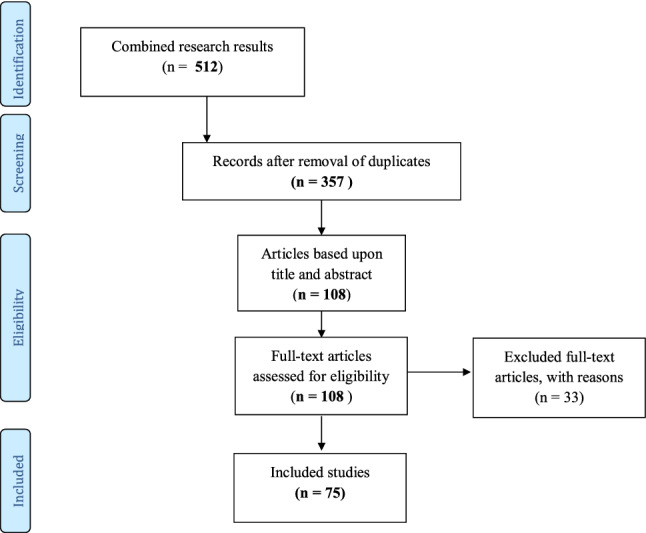
Search process using PRISMA Scoping Review of Literature

In accordance with the indications of Pham and colleagues (Pham et al., 2014), the Preferred Reporting Items for Systematic Reviews and Meta-Analyses (PRISMA) was used to frame the research (see Fig. 1). The combined research results of the 5 databases resulted in 512 records. After de-duplication (using Endnote), 357 records remained which were screened based on title and abstract. Studies that focused on DSD in non-human animals or on gender dysphoria, transidentity, transgender experiences, or transgender persons were excluded. Records that discussed cases of misdiagnosis of DSD were excluded. The same holds true for papers which were published in more sociological, legal or human rights journals. Disagreements between the two reviewers were evaluated by a third person. After the first screening process, 108 records remained. The reference list of the 108 papers was checked to identify any additional studies. No records were added to the final sample. In the next phase, the first author read the full text versions of the manuscripts. After this process, 33 articles were excluded because (1) the primarily focus was not on DSD; (2) they focused on genetic or chromosomal disorders in general; (3) they did not contain any reference to “normal” or “normality” in the full text. The last author verified accuracy and completeness on a 10% sample (see Fig. 1).

The extraction framework contained the following general study characteristics: author names, year of publication, country of origin, journal name, study design, and aim of study, professional background of authors. In order to map data concerning the use of “normal” in the medical care of patients with DSD or a variation of sex characteristics, the team decided to collect also the following information: DSD type, terminology (i.e. DSD, intersex, difference, disorder), and use of normal(ity). Other unanticipated but recurrent thematic data, such as the use of the terms “hermaphrodite” and “ambiguity”, and the inclusion of photographs/illustrations were also added.

## Results

### General characteristics of included studies

A total of 75 papers were included in the review. The vast majority were observational design studies (N 60), in particularly case reports (N = 30, of which 14 on infants or children, 9 on adolescents and 7 on adults), case report series including 2 patients or more (N = 9), prospective (N = 8), and retrospective studies (N = 12). There were also several theoretical papers (N = 12) which focused generally on the DSD spectrum as a whole or discussed various syndromes simultaneously. Only a few of them focused on one specific variation. The sample included one clinical study/experiment (Engeli et al., 2017), one quantitative study which consisted in a survey-based study with 82 pediatric endocrinologists from 15 Arab countries (Deeb et al., 2019). The sample also comprised one qualitative paper on semi-structured interviews with 32 healthcare professionals working in multidisciplinary teams and with experience in treating vaginal agenesis (Roen et al., 2018) and one mixed-methods paper (Dear et al., 2019) based on medical records and a survey with 137 women with untreated vaginal agenesis reporting on their sexual experiences. The latter two studies (which did not have an immediate clinical focus, but concentrated more on attitudes and experiences) were both published in the *Journal of Pediatric and Adolescent Gynecology*. Only 3 out of 75 papers had first or last authors who were non-clinicians, but had a background in psychology (Dear et al., 2019; Roen et al., 2018) or were patient or parent representatives (Dahlmann & Janssen-Schmidchen, 2019). (see Tables 2, 3, 4, 5, 6)

**Table 2:** List of included full texts 2016

References	Country	Journal	Type of study	DSD type	Terminology
Barham et al., (2016)	Hawai	Journal of Urology	CR (infant)	46, XX/46, XY Ovotesticular DSD	Disorder (DSD) "previously known as true hermaphroditism
Carsote et al., (2016)	Romania	Archives of endocrinology and metabolism	CR (adult)	46 XY testicular regression	Intersexuality
Deeb et al., (2016)	United Arabs Emirates	J Clin Res Pediatr Endocrinol	CRS	Deficiency of steroid 5-alpha reductase-2 (5ARD2)	Disorder (DSD)
Fabbri et al. (2016)	Brazil	Sexual Development	CRS	46XY partial gondal dysgenesis	Disorder (DSD)
Hernandez et al. (2016)	Venezuela	Journal of obstretrics and gynaecology	CR (adult)	46 XY Ovotesticular DSD	Disorder (DSD)
Hunter et al., (2016)	USA	Journal of Pediatric and Adolescent Gynecology	CR (adolescent)	46 XY testicular regression	Disorder (DSD)
Larios et al. (2016)	Columbia	Urologia Columbiana	CR (adultI	CAIS	Disorder (DSD)
Lazier et al. (2016)	Canada	American Journal of Medical Genetics	CR (child)	46XY PMDS DSD	Disorder (DSD)
Lepais et al., (2016)	France	Modern Pathology	Retrospective observational study	Mixed	Disorder (DSD)
Marei et al., (2016)	Egypt	Journal of Pediatric Urology	Prospective observational study	CAH	Disorder (DSD)
Mendonca et al., (2016)	Brazil & USA	Journal of Steroid Biochemistry and Molecular Biology	Theoretical	Steroid 5α-reductase 2 deficiency	Disorder (DSD)
Mondal et al., (2016)	India	Indian Journal of Pediatrics	Prospective, observational study	Mixed	Disorder (DSD)
Rich et al., (2016)	USA	Environmental Health Insights	Theoretical	General	Intersex variation and between brackets (hermaphrodite, pseudohermaphroditism)
Simsek et al. (2016)	Turkey	Journal of Clinical Research in Pediatric Endocrinology	CRS	46 XX Ovotesticular DSD	Disorder (DSD)
Singh et al., (2016)	India	International Journal of Infertility and Fetal Medicine	CR (adult)	46 XY complete gonodal dygenesis (Swyer syndrome)	Disorder (DSD)
Tambo et al., (2016)	Cameroon, CH, France	African Journal of Paediatric Surgery	CRS	Mixed gonadal dysgenesis	Disorder (DSD)
Teklu et al., (2016)	Ethiopia	Ethiopian medical journal	CRS	AIS	Intersex disorder

**Table 3:** List of included full texts 2017

References	Country	Journal	Type of study	DSD type	Terminology
Baskin (2017)	USA	Seminars in Perinatology	Theoretical	CAH	Disorder (DSD) they mention controversy
Choudan et al. (2017)	USA	Urology	CRS	46 XX ovotesticular DSD	Disorder (DSD)
Engeli et al., (2017)	CH/Egypt	Journal of Sexual Medicine	Clinical study (experiment)	46XY DSD (general)	Disorder (DSD)
Greeley et al. (2017)	USA	Hormone Research in Paediatrics	CR (infant)	46 XX Ovotesticular DSD	Disorder (DSD)
Jayanthi et al., (2017)	USA	Journal of Pediatric Urology	Retrospective observational study	Proximal hypospadias	Disorder (DSD)
Kearsey et al. (2017)	Australia	Pediatric Surgery International	Theoretical	General	Disorder (DSD)
Khare et al., (2017)	India	Alexandria Journal of Medicine	CR (adolescent)	46,XY complete gonadal dysgenesis (Swyer syndrome)	Disorder (DSD)
Matsumoto et al., (2017)	Japan	Urology	CR (adolescent)	45X/47XYY	Disorder (DSD)
Mazen (2017)	Egypt	Archives of Sexual Behavior	Theoretical	Mixed	Intersexuality and DSD (disorder); no mention is made of the discussion
Poyrazoglu et al., (2017)	Different EU	Journal of Clinical Endocrinology & Metabolism	Retrospect. observational study	Mixed	Disorder (DSD)
Raveenthiran (2017)	India	Indian Journal of Pediatrics	Theoretical	Mixed	Intersex disorder
Sperling et al. (2017)	Germany	Urologe	CR (infant)	PMDS (46 XY DSD)	Disorder (DSD)
Teasdale et al. (2017)	Australia	Journal of Pediatric Endocrinology and Metabolism	CR (infant)	CAH	Disorder (DSD)
Wu et al., (2017)	China	Sexual Development	CRS	45,X/46,XY mosaicism mixed gonadal dysgenesis	Disorder (DSD)

**Table 4:** List of included full texts 2018

References	Country	Journal	Type of study	DSD type	Terminology
Berberoğlu et al. (2018)	Turkey	JCRPE Journal of Clinical Research in Pediatric Endocrinology	Retrospective observational study	46 XY and 45 X/46,XY Mixed Gonodal dysgenis	Disorder (DSD)
Chen et al., (2018)	Taiwan	Urological Science	Retrospective observational study	Mixed	Disorder (DSD)
Chowdhury et al., (2018)	Bangladesh & USA	International Journal of Surgery Case Reports	CR (child)	46 XY 5-alpha reductase deficiency	Disorder (DSD)—previously "intersex" disorders
Heo et al., (2018)	South-Korea	Annals of Pediatric Endocrinology and Metabolism	CRS	CAIS	Disorder (DSD)
Jesus et al., (2018)	Brazil	Journal of Pediatric Surgery	Retrospective observational study	Mixed	Disorder (DSD)
Johannsen et al. (2018)	Denmark	Journal of Clinical Endocrinology & Metabolism	Prospective observational study	Mixed	Disorder (DSD)
Kornman et al., (2018)	Australia	Fetal Diagnosis and Therapy	Prospective observational study	general	Disorder (DSD)
López-Hernández et al. (2018)	Mexico	Reproductive BioMedicine Online	Retrospective observational study	46,XX ovotesticular DSD	Disorder (DSD)
Mirshahvalad et al., (2018)	Iran	Urology	CR (adolescent)	46 XX Ovotesticular DSD	Disorder (DSD)
Morandi et al., (2018)	UK	Journal of Clinical Endocrinology & Metabolism	CR (longitudinal child)	45,X/46,XY mosaicism mixed gonadal dysgenesis	Disorder (DSD)
Morozumi et al., (2018)	Japan	Tohoku Journal of Experimental Medicine	CR (child)	46 XY DSD	Disorcer (DSD)
Roen et al., (2018)	Norway/UK	Journal of Pediatric and Adolescent Gynecology	Qualitative study	Mixed	“disorder”
Roth et al., (2018)	USA	Urology Case Reports	CR (child)	Ovotesticular DSD 46XX/XY	Disorder (DSD)
Witchel. (2018)	USA	Best Practice and Research: Clinical Obstetrics and Gynaecology	Theoretical	General	Disorcer (DSD)
Xu et al., (2018)	China/USA	Journal of Pediatric Endocrinology and Metabolism	CR (child)	46 XY Leydig cell hypoplasia (LCH)	Disorder (DSD)
Yan et al. (2018)	China/USA	Journal of Clinical Research in Pediatric Endocrinology	CR (child)	46 XY Leydig cell hypoplasia (LCH)	Disorder (DSD)

**Table 5:** List of included full texts 2019

References	Country	Journal	Type of study	DSD type	Terminology
Ameyaw et al., (2019)	Ghana, UK, Australia, Canada	Archives of Disease in Childhood	Prospective incidence study	Mixed	Disorder (DSD)
Caputo et al., (2019)	Italy	Urology	CR (adult)	45X/46XY Ovotesticular DSD	Disorder (DSD)
Dahlmann et al. (2019)	Germany	Monatsschrift fur Kinderheilkunde	Theoretical	CAH	Difference/Disorder (DSD), intersex, variations & reference to discussion
Dear et al., (2019)	UK	Journal of Pediatric and Adolescent Gynecology	Mixed methods	Mixed	DSD/intersex
Deeb et al., (2019)	UAE, Egypt, Jordan, Algeria	Journal of Pediatric Endocrinology and Metabolism	Quantitative	Mixed	Disorder (DSD)
Finlayson et al., (2019)	USA	Journal of the Endocrine Society	Prospective observational study	CAH	Difference/Disorder (DSD) but no discussion of terminology
Goncalves et al., (2019)	USA	Seminars in Pediatric Surgery	Theoretical	CAH	Disorder (DSD)
Hertweck et al. (2019)	USA	Journal of Pediatric and Adolescent Gynecology	Theoretical	Mixed	Disorder (DSD) but also intersex as a key word
Huang et al., (2019)	Taiwan	Journal of the Formosan Medical Association	Retrospective observational study	45,X/46,XY Mosaicism DSD mixed gonadal dysgenis	Disorder (DSD)
Miller (2019)	USA	Hormone Research in Paediatrics	Theoretical	CAH	Disorder (DSD)
Nasir et al., (2019)	Nigeria	Journal of Pediatric and Adolescent Gynecology	Prospective observational study	Mixed	Disorder (DSD)
Ochi et al., (2019)	Japan	Pediatric Surgery International	Retrospective observational study	General	Disorder (DSD)
Özdemir et al., (2019)	Turkey	Journal of clinical imaging science	CR (young adult)	Ovotesticular 46 XX DSD	Disorder (DSD)
Pan et al., (2019)	China	BMC Pediatrics	Retrospective observational study	45,X/46,XY mosaicism (mixed)	Disorder (DSD)
Patil et al., (2019)	India	Journal of Obstetrics and Gynecology of India	CR (young adult)	CAH	Disorder (but not DSD)
Pranckėnienė et al., (2019)	Lithuania	Journal of Pediatric and Adolescent Gynecology	CR (adolescent)	AIS	Disorder (DSD)
Raveendran et al., (2019)	India	Andrologia	CR (adolescent)	46,XY complete gonadal dysgenesis	Disorder (DSD), formerly termed intersex conditions
Saikia et al., (2019)	India	Journal of Human Reproductive Sciences	CR (child)	mixed gonadal dysgenesis (45X/47 XYY)	Disorder (DSD)
Schteingart et al., (2019)	Argentina, France, USA	Human Molecular Genetics	CR (child)	46 XY PMDS SDSD	Disorder (DSD)
Touzon et al., (2019)	Argintina	JCRPE Journal of Clinical Research in Pediatric Endocrinology	Prospective observational study	AIS	Disorder (DSD)
Van Leeuwen et al., (2019)	USA	Seminars in Pediatric Surgery	Retrospective observational study	mixed	Disorder (DSD)
Wagner-Mahler et al., (2019)	France	Molecular Genetics and Genomic Medicine	CR (adolescent)	46 XY gonodal dysgenesis	Disorder (DSD)
Weaver et al., (2019)	USA	Urology	CR (child)	46 XY gonodal dysgenesis	Disorder (DSD)
Weidler et al. (2019)	USA	Seminars in Pediatric Surgery	Theoretical	General	Differences of sexual development (DSD)—mention debate surrounding terminology

**Table 6:** List of included full texts 2020

References	Country	Journal	Type of study	DSD type	Terminology
Abouzeid et al. (2020)	Egypt	Journal of pediatric surgery	Retrospective observational study	CAH	Disorder (DSD)
Adriao et al. (2020)	Portugal	Clinical Pediatric Endocrinology	CR (adolescent)	46 XX testicular DSD (Chapelle syndrome)	Disorder (DSD)
Fadil et al. (2020)	Argentina	Revista Chilena de Pediatria	CRS	Klinefelter	Disorder (DSD)
Pattamshetty et al., (2020)	India	Journal of reproduction & infertility	CR (adolescent)	Klinefelter syndrome mosaicism 46,XX/47,XXY	Disorder (DSD)

Most papers came from the following regions: Asia–Pacific (N = 20), North-America (N = 16), the EU (N = 11), Middle-East (N = 8) and Latin-America (N = 7). Africa (N = 3) was largely underrepresented in the review. Ten papers came from more than one country. The medical journals in which the articles were published focused mainly on gynecology, reproduction, obstetrics & perinatology (N = 18); endocrinology (N = 14); urology (N = 11), surgery (N = 6), and to a lesser extent on genetics (N = 3), clinical research (N = 2) and environmental health (N = 1). The most common discussed forms of DSD were: ovotesticular DSD (N = 10); congenital adrenal hyperplasia (CAH (N = 9), mixed gonadal dysgenesis (N = 6); Androgen Insensitivity Syndrome AIS (N = 5); and complete gonadal dysgenesis (N = 3). Fifteen articles discussed various forms of DSD together and five papers did not address any syndrome in particular (see Tables 2, 3, 4, 5, 6).

### Key terms and concepts with regard to DSD/intersex

With the exception of very few papers, all studies used the **acronym DSD** as proposed by the *Chicago Consensus Statement* of 2006 to indicate congenital conditions in which development of chromosomal, gonadal or anatomical sex is atypical. Moreover, there was wide consensus to interpret the first part of the abbreviation as “**disorder**” rather than as difference or diversity. Only 3 (Dahlmann & Janssen-Schmidchen, 2019; Finlayson et al., 2019; Weidler & Peterson, 2019) out of 75 papers, in fact, used the expression “**differences of sexual development**” next to “disorder” and 3 papers (Baskin, 2017; Dahlmann & Janssen-Schmidchen, 2019; Weidler & Peterson, 2019) reported that the medical terminology DSD remains controversial. The study of Lepais and colleagues criticized the new DSD classification system for not taking into account histological anomalies (Lepais et al., 2016). Five studies used a term derived from **intersex**: intersexuality (Carsote et al., 2016; Mazen, 2017), intersex variation (Rich et al., 2016), intersex condition (Raveendran et al., 2019) or intersex disorder (Chowdhury et al., 2018; Raveenthiran, 2017; Teklu et al., 2016), not in the sense of an identity to be proud of, but rather as a more outdated medical concept for physical sex ambiguities. One paper (Roen et al., 2018) expressed its critique on the term “disorder” by placing it between hyphens. The word **hermaphrodite** was mentioned in 10 of 75 papers (Barham et al., 2016; Caputo et al., 2019; Greeley et al., 2017; Hernández et al., 2016; Mirshahvalad et al., 2018; Özdemir et al., 2019; Rich et al., 2016; Schteingart et al., 2019; Şimşek et al., 2016; Yan et al., 2019), often in the context of ovotesticular DSD. Five papers used the outdated term to indicate that this condition was “previously known” as hermaphroditism (Barham et al., 2016; Caputo et al., 2019; Özdemir et al., 2019; Rich et al., 2016; Şimşek et al., 2016), 4 studies used it as a mere synonym (Greeley et al., 2017; Mirshahvalad et al., 2018; Schteingart et al., 2019; Yan et al., 2019) and only one article explicitly stated that the term has been replaced because it was considered stigmatizing (Hernández et al., 2016) (see Tables 2, 3, 4, 5, 6).

### The role of medical photography and other visual images

A total of 29 records (38.7%) included medical photographs of the patient’s body or body parts. In most cases, they were close-ups of genitals—mainly from **infants or children** (AbouZeid & Mohammad, 2020; Barham et al., 2016; Baskin, 2017; Deeb et al., 2016; Fadil Iturralde et al., 2020; Goncalves et al., 2019; Heo et al., 2018; Jayanthi et al., 2017; Jesus et al., 2018; Kearsey & Hutson, 2017; Marei et al., 2016; Matsumoto et al., 2017; Morozumi et al., 2018; Nasir et al., 2019; Ochi et al., 2019; Saikia et al., 2019; Şimşek et al., 2016; Sperling & Meyer, 2017; Wagner-Mahler et al., 2019), but in a few cases also of **adolescents** (Chouhan et al., 2017; Hunter et al., 2016; Tambo et al., 2016) and **adults** (Larios García & Bautista Delgado, 2016; Patil et al., 2019; Singh et al., 2016). Three papers also contained images of **breasts** (Singh et al., 2016) **and/or fully body shots** (Khare et al., 2017; Mirshahvalad et al., 2018) of adolescents whose eyes were obscured with a black line. One paper contained images of the genitals of both children and adolescents (Raveenthiran, 2017). Most of the 29 papers were case studies, but there were also 4 theoretical studies (Baskin, 2017; Goncalves et al., 2019; Kearsey & Hutson, 2017; Raveenthiran, 2017) and one survey study (Deeb et al., 2019). One paper focused on the use of imaging techniques in the identification of DSD (Goncalves et al., 2019). Medical photographs were used over the full 2016–2020 interval. None of the studies explicitly reported having obtained informed consent from parents or patients to publish these photos. When informed consent was mentioned it referred to consent to participation in the study. None of papers discussed the unique ethical implications of using medial photographs for publication purposes.

Only 2 papers (Marei et al., 2016; Baskin, 2017) included illustrations or so-called schematic representation of respectively (1) vaginal depth; and (2) the anatomy of a female with CAH.

### The use of “normal” or “normality”

**Fig. 2:**
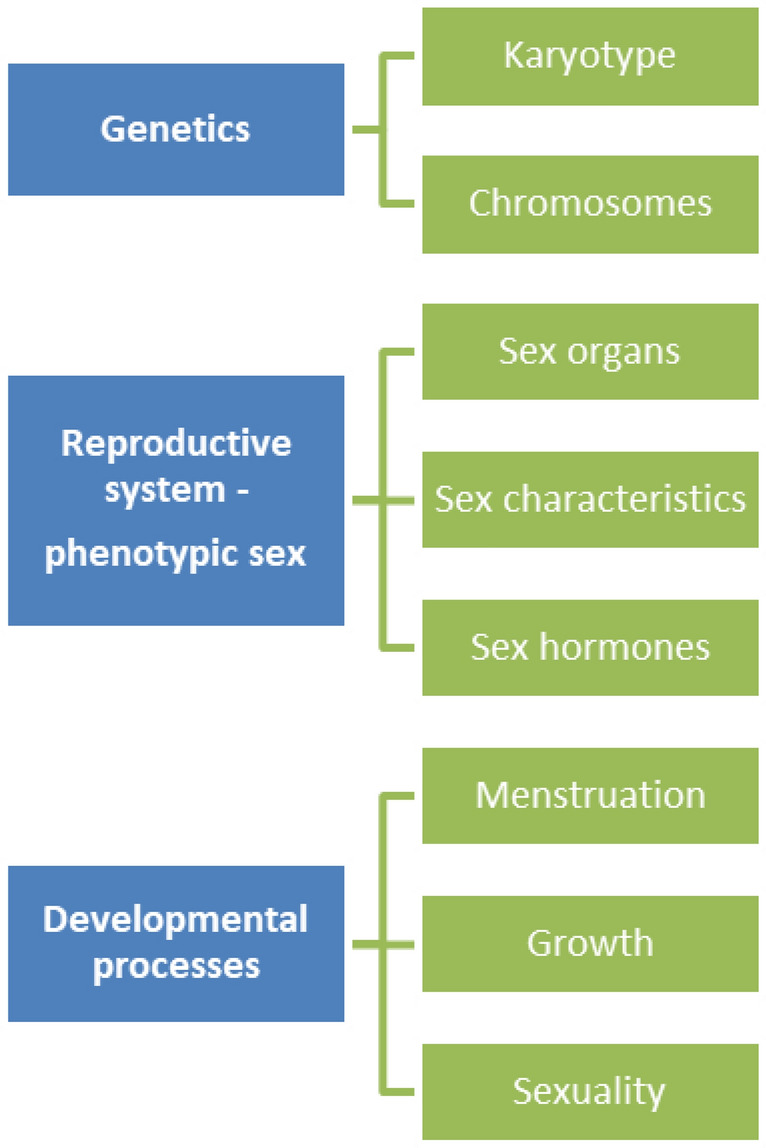
Uses of normal-normality I

The term ‘normal’ or ‘normality’ and their opposites, ‘abnormal’ or ‘abnormality’ were frequently used throughout the papers. Normal was frequently used in relationship to **genetics**, and in particular to karyotype (e.g. normal karyotype, normal male karyotype) and chromosomes (e.g. normal chromosomes, abnormalities in sex chromosomes (= mosaicism), chromosomal abnormalities) (AbouZeid & Mohammad, 2020; Fadil Iturralde et al., 2020; Heo et al., 2018; Hernández et al., 2016; Lepais et al., 2016; Matsumoto et al., 2017; Morandi et al., 2018; Rich et al., 2016; Tambo et al., 2016; Wagner-Mahler et al., 2019; Weaver et al., 2019). A karyotype is the visual depiction of all the chromosomes in a cell which generally contains 23 pairs of chromosomes of which 22 pairs are called autosomes and one pair are the so-called sex chromosomes (e.g. XX for genotypic female and XY for genotypic male) (see Fig. 2).

The most common use of normal was in association with **phenotypic sex** (e.g. normal male or female phenotype). In one case, however, reference was made to phenotypical men (Wu et al., 2017). The great majority of the included papers made usage of normal to refer to the individual’s **reproductive system**, and in particular to primary and secondary internal and external **sex organs** (e.g. normal vagina, normal penis, normal ovaries, normal testes, normal labia, normal ovarian tubes, normal fallopian tubes, abnormality of scrotum). Only in some occasions did the authors explain in more detail why these organs were considered (ab)normal, namely in terms of **appearance** (Adrião et al., 2020; Barham et al., 2016; Chen et al., 2018; Chowdhury et al., 2018; Fadil Iturralde et al., 2020; Hernández et al., 2016; Nasir et al., 2019; Ochi et al., 2019; Özdemir et al., 2019; Patil et al., 2019; Roth et al., 2018; Schteingart et al., 2019; Weaver et al., 2019), **size** (Adrião et al., 2020; Marei et al., 2016; Matsumoto et al., 2017; Mondal et al., 2016; Rich et al., 2016; Schteingart et al., 2019), **length** (AbouZeid & Mohammad, 2020; Finlayson et al., 2019; Goncalves et al., 2019; Morandi et al., 2018; Raveenthiran, 2017; van Leeuwen et al., 2019), **location** (Finlayson et al., 2019), and **function** (Heo et al., 2018; Lepais et al., 2016; Poyrazoglu et al., 2017; Raveendran et al., 2019; Raveenthiran, 2017). Some examples include: normal looking genitalia, normal appearing uterus, normal penis size, abnormal enlargement of clitoris, vagina with normal length, normally descended testes, abnormal location of the urethral meatus, and normal testicular function. Overall, appearance was the most commonly cited criteria of normality for sex organs (see Fig. 2).

Many papers (Caputo et al., 2019; Heo et al., 2018; Hunter et al., 2016; Kearsey & Hutson, 2017; Larios García & Bautista Delgado, 2016; Patil et al., 2019; Poyrazoglu et al., 2017; Raveenthiran, 2017; Rich et al., 2016; Saikia et al., 2019; Schteingart et al., 2019; Şimşek et al., 2016; Singh et al., 2016; Touzon et al., 2019; Wagner-Mahler et al., 2019; Yan et al., 2019) also referred to (ab)normal **hormonal levels** or (ab)normal **hormone production** (e.g. testosterone, estrogen, luteinizing hormone (LH), follicle stimulating hormone (FSH), Anti-Müllerian hormone (AMH)), at times with the indication of a reference range (Adrião et al., 2020; Morandi et al., 2018; Raveendran et al., 2019; Şimşek et al., 2016; Sperling & Meyer, 2017; Teasdale & Morton, 2017; Wu et al., 2017; Xu et al., 2018). A few articles (Heo et al., 2018; Khare et al., 2017; Larios García & Bautista Delgado, 2016; Mirshahvalad et al., 2018; Özdemir et al., 2019; Pranckėnienė et al., 2019) also referred to **secondary sex characteristics** such as breasts (e.g. normal breast development) and hair (e.g. normal hair growth, normal pubic hair development, normal facial and axillary hair). Given that these secondary sex features mark adult maturation, also **pubertal processes** were regularly cited in the literature (Fabbri et al., 2016; Greeley et al., 2017; Heo et al., 2018; Hernández et al., 2016; Hertweck & Rothstein, 2019; Huang et al., 2019; Hunter et al., 2016; Kearsey & Hutson, 2017; Mirshahvalad et al., 2018; Morandi et al., 2018; Özdemir et al., 2019; Pranckėnienė et al., 2019; Schteingart et al., 2019). Examples include: normal pubertal development, normal puberty, normal growth, normal menstruation, abnormal virilization, normally masculinized males, sexually abnormal, normal fertility, reproductive abnormalities, normal fertility potential) (see Fig. 2).

**Fig. 3:**
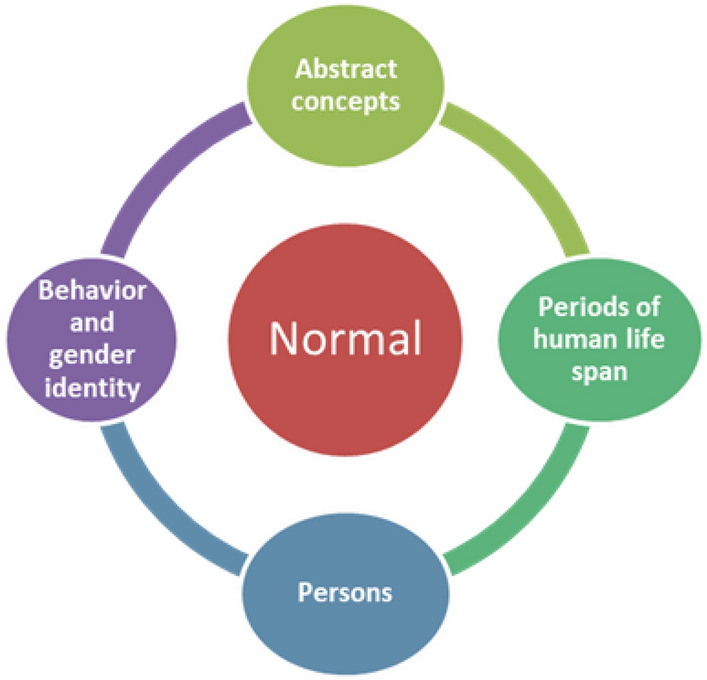
Uses of normal-normality II

In some papers (AbouZeid & Mohammad, 2020; Caputo et al., 2019; Chouhan et al., 2017; Heo et al., 2018; Hernández et al., 2016; Hertweck & Rothstein, 2019; Larios García & Bautista Delgado, 2016; Marei et al., 2016; Miller, 2019; Poyrazoglu et al., 2017; Pranckėnienė et al., 2019; Rich et al., 2016; Teasdale & Morton, 2017; Wu et al., 2017) normal was also employed to designate more **abstract concepts** (e.g. normal quality of life, normal sexual life, normal sex satisfaction), **periods of the human life span** (e.g. normal childhood), **persons** (e.g. normal infant, normal neonate, normal child, normal boy, normal individual, normal patient) and **behavior & gender identity** (e.g. normal behavior, normal gender development) (see Fig. 3). None of the papers provided any definition or description of what normal meant in these cases. With regard to sexual life, gender identity, behavior and development implicit references to heteronormativity were common.

Finally, two papers (Dear et al., 2019; Roen et al., 2018) placed the words “normal” or “normality” between hyphens and warned for the normative power its use might have upon the care and self-perception of women with vaginal agenesis (see Table 7).

**Table 7:** Uses of “normal” or “normality”

Critical reflection on the normative power of “normal” or “normality”	Certain ways of talking about bodily difference can imply a “need” for treatment, which can increase the emotional burden of normative pressure. It might be more helpful to avoid medicalizing vocabularies when discussing bodily and sexual matters. So doing introduces broader factors other than vaginal size and genital intercourse to affect the women's self-understandings and opens the door for questioning what is “normal” in sex (…) Multidisciplinary teams (MDTs) would need to proactively question social norms about how women's genitals should appear and function. Second, and complementing the first suggestion, MDTs need to actively transform the clinical focus from treatment to women (Roen et al., 2018, 250–251)
	(…) the pressure to normalize” might result in hasty treatment decisions, unrealistic expectations, and inadequate preparation, resulting in despondency and disengagement (…). If the focus of care were to be transformed to relationship and sexual confidence rather than vaginal size, when counseling women, health professionals might wish to emphasize sensual exploration, emotional intimacy, and sexual pleasure rather than coital performance (Dear et al., 2019, 301 and 304)

### Terminology and clinical care

The studies touched on some important DSD-specific themes such as: (delay in, correct, timely etc.) diagnosis (Ameyaw et al., 2019; Caputo et al., 2019; Fadil Iturralde et al., 2020; Mondal et al., 2016; Şimşek et al., 2016; Teasdale & Morton, 2017; Xu et al., 2018), medical decision-making (Hernández et al., 2016; Khare et al., 2017; Teasdale & Morton, 2017; Witchel, 2018), multi-disciplinary teams (Berberoğlu & Şıklar, 2018; Caputo et al., 2019; Chowdhury et al., 2018; Heo et al., 2018; Hernández et al., 2016; Kearsey & Hutson, 2017; Nasir et al., 2019; Özdemir et al., 2019; Şimşek et al., 2016; Witchel, 2018; Wu et al., 2017) follow-up care (Berberoğlu & Şıklar, 2018; Chouhan et al., 2017; Hertweck & Rothstein, 2019), genetic screening (Kornman et al., 2018; Wu et al., 2017), fertility and reproduction (Adrião et al., 2020; Greeley et al., 2017; Hertweck & Rothstein, 2019; Pattamshetty et al., 2020; Witchel, 2018), the risk for tumor development of gonads (Chen et al., 2018; Chouhan et al., 2017; Heo et al., 2018; Huang et al., 2019; Lepais et al., 2016; Matsumoto et al., 2017; Pan et al., 2019; Şimşek et al., 2016) and sexuality (Baskin, 2017; Mendonca et al., 2016; Patil et al., 2019).

There was no direct link between the use of normal/normality and medical interventions insofar some studies recommended early surgeries (Barham et al., 2016; Baskin, 2017; Deeb et al., 2019; Greeley et al., 2017; Hernández et al., 2016; Huang et al., 2019; Jayanthi et al., 2017; Khare et al., 2017; Larios García & Bautista Delgado, 2016; Matsumoto et al., 2017; Wagner-Mahler et al., 2019; Xu et al., 2018) and others suggested to postpone them and were more hesitant/conservative regarding gonadectomy (AbouZeid & Mohammad, 2020; Berberoğlu & Şıklar, 2018; Chen et al., 2018; Morandi et al., 2018; Ochi et al., 2019; Sperling & Meyer, 2017; Witchel, 2018). To illustrate this, we have selected a few quotes from the reviewed literature.

In a few cases, surgery was explicitly justified in the name of normality (appearance, functioning, life) (see Table 8; I.a.). In other cases, surgery was recommended in reference to risk of malignancy and parental wishes (see Table 8; I.b.).

**Table 8:** Terminology and clinical care

I. Justification of surgery	
I.a. Normal appearance	Based on expert opinion, the overwhelming majority of surgical specialists continue to recommend restoration of normal anatomy in female patients in early childhood (Baskin et al. 2017, p. 230) A 2-year-old child reared as a girl child was brought by parents with ambiguous genitalia noticed since birth (…) the child underwent bilateral gonadectomy and rehabilitated her to lead a life as a girl (Saikia, 2019, p. 169)
I.b. Risk of malignancy	This case highlights the importance of thorough surgical exploration to ensure full excision of all gonadal tissue discordant with sex of rearing (…) Goals of surgery include achieving desirable cosmesis, establishing urinary function, preserving any future fertility, and eliminating or decreasing the risk of malignancy (Barham, 2016, p. 195) Some recent studies have recommended gonad removal after puberty. In our patients, the gonads were removed before puberty reflecting the wishes of the parents to escape confusion for the patients’ gender identity (Heo, 2018, p. 224)
I.c. Questioning risk of malignancy	Growing evidence suggested the frequency of gonadoblastoma in DSD patients remains various and it is not necessary for all DSD patients to receive gonadectomy. Moreover, the optimal timing of gonadectomy remains controversial. The rarity of the disorder makes the establishment of standard treatment guidelines difficult. The individualized treatment strategy is very important for each patient with DSD (…) the preference of patients and parents should be put into consideration (Chen et al., 2018, pp. 243–244) The gonadal tumor risk is highly variable in disorders of sex development, (…) Whereas early gonadectomy and feminizing surgery were often recommended in disorders of sex development with a high tumor risk the current attitude is to avoid mutilating surgeries in children and to identify more accurately the tumor risk of gonads (Lepais 2016, p. 1400)

II. Referral of surgery	
II. a Stigmatizing/heteronormative language	Approximately 1 in 20,000 “female children” born is actually a male with complete androgen insensitivity syndrome (CAIS). In normal females, traces of androgen secreted from adrenals will effect minimal masculinization such as hairy legs and acne. But individuals with CAIS will be fully feminized with typical female phenotype sans uterus and vagina. They even develop attractive breasts at puberty (…) Therefore, it is appropriate to raise CAIS boys as girls. Vaginoplasty and perhaps orchidectomy may be postponed until late adolescence (Raveenthiran, 2017, p. 704) Removal of testes in patients with complete androgen insensitivity is controversial. Most recent data indicate that the risk for tumor development is low until early adult years (…) Delayed surgery promotes shared decision-making with the patient, family, and healthcare providers (…) No uniform consensus regarding the indications, timing, and extent of the operation is applicable for individuals with DSDs. Each patient warrants individual contemplation and attention by a multidisciplinary team at experienced centers. Considerations include future fertility, risk for gonadal tumors, propensity for urinary tract infections, avoiding stigmatization related to atypical genital anatomy, and ensuring functional genital anatomy to allow future penetrative intercourse (Witchel, 2018, p. 97)

III. Medical language & attitudes	
III.a. Critical reflection	Far from assuming that untreated women cannot “have sex” because they could not physically engage in coitus, our findings suggest that it is important to recognize the women as sexual beings regardless of their genital anatomy. Instead of introducing vaginal construction as something that the women (will) need to “have sex,” health professionals should emphasize to the women that they are already able to access sexual intimacy, relationships, and pleasure. In situating vaginal construction as a choice within a care pathway with a much broader focus, care providers can avoid exacerbating normative pressures on women to take up vaginal construction (too soon) (Dear et al., 2019, p. 303) In many cases, reproductive and DSD diagnoses are complex and might require the clinician to help disclose age appropriate information to the patient regarding the pathophysiology of her reproductive condition avoiding medical jargon (…) For example, when providing this information, it is important to avoid confusing terminology such as “blind vagina” in the cases of vaginal agenesis (Hertweck et al. 2019, p. 108)

IV. Patients’ lived experience	
IV.a. Biomedical approach	We present an interesting; almost bizarre case of a male with non-functional testes early during childhood and undiagnosed and untreated hypogonadism until his fifth decade of life. We checked the grip strength of our patient and recorded abnormally low levels for the male sex, within the normal ranges for a woman of the same age. Interestingly in this case the patient had no sexual activity, preferences, sexual interest or needs probably due to the effects of early testosterone deficiency on brain development (Carsote et al., 2016, p. 80)
IV.b. Psychological evaluation	The present case [of ovotesticular DSD] is an extremely rare one in that the external genitalia is nearly normal, and the patient (and his parents) has not been in doubt about any sexual abnormality until a progressive breast enlargement occurred. We think that this delay in applying for medical care might be an unconscious resistance to a possible diagnosis of a sexual abnormality. In our patient, reactive behaviors such as not believing in the diagnosis and not cooperating in treatment processes were observed at the first. After accepting the diagnosis, he developed symptoms of severe depression (Özdemir, 2019, p. 4) The genetic findings were explained to the patient [a 16-year-old Hispanic female adolescent], who expressed female gender identity and frustration with her lack of development of secondary sexual characteristics. Laparoscopy with gonadectomy was recommended (Hunter 2016, p. e61)
IV.c. Patient-centered approach	A previously healthy 13-yr-old male adolescent was referred to the pediatric endocrinology outpatient clinic due to progressive bilateral gynecomastia. This feature was a major concern for the adolescent during the previous year (…) In this particular case, due to azoospermia and the risk of gonadoblastoma associated with the 46, XX male disorders of sexual development and very small testicles, surgical removal and testicular prothesis were recommended to the adolescent, which is still under consideration. Meanwhile, self-examinations and regular ultrasound were advised (Adriao, 2020, pp. 43–44)

V. The role of culture and religion	
V.a. Impact on medical decisions	Ten percent of our study participants recommended surgery at puberty or no surgery. This is a relatively low percentage considering the emerging trend of deferring surgery to adulthood with consideration of involving patients in decision-making (…) cultural and religious factors do have an impact on gender assignment leading to unique practice in the Islamic region irrespective of whether international consensus guidelines are available or lacking (Deeb, 2019, pp. 80–81) Working in a largely conservative and poorly resourced African population implies that decisions on management need to reflect the importance of the family unit, the customs, and the lack of social services (…) the upbringing in these communities is communal-based, an early decision on the gender of rearing is essential and assists early integration of the child into the society (…) Open communication might help parents of young children make informed decisions that are in line with their personal and cultural values leading to greater confidence in decision-making with greater satisfaction and less regret (Nasir et al., 2019, p. 26)

Several papers (Chen et al., 2018; Lepais et al., 2016) criticized the validation of surgery by referring to malignancy and highlighted that gonadal tumor risk is highly variable depending on the DSD syndrome and hence, expressed the need for an individualized approach (see Table 8; I.c.). Among the studies that were more conservative with regard to early surgery, some used nevertheless very normalizing and stigmatizing language. In the cited example, the authors used hyphens to question the child’s sex, took on a very binary and genetically determined view on sex and used attractiveness of secondary sex characteristics to determine sex of rearing (see Table 8; II.a.).

In another study (Witchel, 2018), the authors seem to be in favor of deferring surgery in some DSD variations and to emphasize the need for shared decision-making with patients and families and patient-centered care, but on the other hand they seem to cast persons with DSD as being in need of expert knowledge and treatment and to use rather heteronormative language, especially in reference to sexuality. Only a few studies (Dear et al., 2019; Hertweck & Rothstein, 2019) expressed concern about how healthcare professionals’ attitudes and language use can impact patient’s self-perception and decision-making (see Table 8; III a).

Apart from the study of Dear and colleagues (Dear et al., 2019), published in the *Journal of Pediatric and Adolescent Gynecology*, and the theoretical study of Dahlman et al. (Dahlmann & Janssen-Schmidchen, 2019) on the viewpoint of patient organizations on early genital surgery, the great majority of the studies presented the viewpoint of healthcare professionals with little or no attention for persons’ lived experience. Only in a few occasions, a brief allusion was made to the patient’s view (Adrião et al., 2020; Hunter et al., 2016). Carsote and colleagues (Carsote et al., 2016), for example, draw attention to what they call a very rare case of a Caucasian man of 46 with DSD. They report about the patient’s absent sexual life, but only refer to the possible biological causes of this lack of interest rather than to his lived experience (see Table 8, IV.a). Other papers (Hunter et al., 2016; Özdemir et al., 2019) sometimes included psychological evaluations of a patient or mentioned their concerns, emotions and frustrations. However, Hunter and colleagues (Hunter et al., 2016) failed to report how the patient was informed about the surgery and what she herself thought about it (see Table 8, IV.b). Also Adriao et al. (Adrião et al., 2020) presented a case study of an adolescent who was brought to the hospital out of concern about the enlargement of his breasts due to the growth of breast tissue. The authors took a more patient-centred approach and mention that the family has not yet consented to surgery which was recommended because of tumor risk (see Table 8, IV.c.).

Finally, only a few papers emphasized the importance of culture and religion in the medical management of DSD (Ameyaw et al., 2019; Deeb et al., 2016, 2019; Marei et al., 2016; Mazen, 2017; Nasir et al., 2019). They highlighted that some DSD variations are more frequent in areas where consanguineous families are more common, pointed out to specific challenges (e.g. delay in diagnosis, lack of essential medicines for salt-wasting CAH), gender roles) and seemed to warn for potential ethnocentric interpretations of existing international clinical guidelines when imposed on non-western countries (See Table 8; V.a).

## Discussion

The pursuit of normality has for long characterized the medical management of persons born with variations of sex characteristics. In this review we aimed to describe how normal/normality is used in the medical literature on DSD or intersex since the publication of the 2016 update to the 2006 DSD Consensus Statement of Chicago and to explore whether the use of normal/normality somehow informs clinical care.

About half of the studies in our sample were case reports on one or more patients and focused on rare forms of DSD variations. Case reports are quite common in the history of medical literature as they allow healthcare professionals to share and transfer their clinical observations, diagnostic approach and knowledge with and to other specialists in the field (Alsaywid & Abdulhaq, 2019). Their ultimate aim is to optimize patients’ quality of care. Given that within medicine, DSD variations are considered a group of rare or uncommon conditions due to their relatively small numbers, rendering cohort studies challenging to perform, the pre-dominance of case studies in our sample should not come as a surprise. If we consider that healthcare professionals in many countries have usually only limited knowledge and understanding of DSD due to the rarity and heterogeneity of these conditions, case studies are of fundamental importance to better understand and improve the care of intersex persons.

The finding of an apparent interest in sharing knowledge about rare conditions, is further supported by the fact that the most commonly discussed variation in our sample (across the various study types) was ovotesticular DSD—a condition characterized by the presence of both ovarian and testicular tissues in the gonads of the same individual—which is among the most uncommon intersex variations.

Case reports consist by definition of a presentation of the patient’s condition written by an expert, but—in line with Sir William Osler’s (Osler, 1925) saying that it is “a safe rule to have no teaching without a patient for a text, and the best teaching is that taught by the patient himself”—the inclusion of patient’s perspectives on their own experiences is increasingly welcomed as an additional, valuable dimension (Alsaywid & Abdulhaq, 2019). However, in the papers we selected, little or no attention was given to patients’ lived experiences. The clinician-centered lens in most studies was further emphasized by the use of the acronym DSD which, in line with the Consensus Statement (Lee et al., 2006) and ICD-11, was most frequently interpreted as disorders of sexual development. Most papers failed to address the controversy surrounding this concept, bypassing the fact that the word “disorder” is incompatible with how many people with variations of sex development identify themselves and risks turning a mere somatic difference into a pathological condition in need of fixing (Lundberg et al., 2018). Even more troublesome was the reference to the archaic word hermaphrodite in more than 13% of the sample (which should maybe not come as a full surprise given its use in the ICD-11). For many affected persons the invocation of this Greek mythological figure is however highly offensive (Hillman, 2008). The dominance of the clinical view in many studies was further highlighted by the frequent use of genital photography and in a few cases even of full body shots, not only in case reports but even in mere theoretical studies. The lack of any critical reflection on the use of such intimate photos for publication reasons is unsettling and risks reducing the person into a mere study object for the medical gaze without the possibility of reciprocating that gaze given the absence of patients’ lived experience. Although the use of illustrations is at first sight less problematic than the use of photos, since it does not seem to imply the risk of objectification, in practice drawings of so-called genital (ab)normality might have the same normative power as photographs and drive treatment practices that aim to fix “doubtful” reproductive organs ((Karkazis, 2010; Wall, 2010).

Critical studies on the biomedical approach to intersex that include experiences of affected persons are not uncommon, but are most of the time not published in medical journals and will therefore likely be missed by clinicians (Jones, 2018). Likewise, social scientists rarely ever engage with medical articles on DSD and tend to publish in social science, and ethics journals. This strong disconnection between these two “worlds” of research is deeply problematic if we want to improve the care of intersex persons. In our sample, 3 articles somehow formed an exception to this trend insofar they were published in medical journals (*Journal of Pediatric and Adolescent Gynecology*; *Monatsschrift fur Kinderheilkunde*), but written by social scientists and clinicians together (Dear et al., 2019; Roen et al., 2018) or by patient and parent representatives (Dahlmann & Janssen-Schmidchen, 2019).

The lack of cross-fertilization between different interdisciplinary approaches to DSD or intersex is maybe even more apparent in the use of normal/normality in the medical literature. Whereas in the field of disability studies, critical race theory, philosophy, gender studies, history and anthropology a critical reflection on the normative power of normality is not new (Cryle & Stephens, 2017); within medicine such debates seem less dominant. The great majority of the papers in our review used this concept quite lightly without much consideration for its impact or meaning, blending descriptive and normative aspects. In the retrieved literature, normal was frequently used in relationship to genetics and seemed to take on the (implicit) meaning of “most commonly encountered” (i.e. 46 XX and 46 XY). This is not unexpected given that the new DSD classification system is grounded in karyotype. The most common use, however was with regard to phenotypic sex. Still, it was not always explicit in what this normality (e.g. normal vagina) consisted (i.e. appearance, size, location, function) and even if it was specified, the question can be raised what, for example, “a normal appearing vagina” means. Does it refer to the most commonly encountered vagina? Studies have shown that there exists a wide variety in the appearance (and size) of women’s and girls’ genitals (Brodie et al., 2019; Lloyd et al., 2005). Moreover, none of the papers that included photographs of “abnormal” genitals included photos of phenotypically “normal” genitals for comparison. Hence, it was unclear why these organs were considered (ab)normal in terms of appearance.

In some cases, normality was used in reference to biological function, which seemed to imply fertility, reproduction, penetrative intercourse. However, as pointed out by the philosopher Ron Amundson (Amundson, 2000, p. 33), functional normality is not supported by current biology and often “the disadvantages experienced by people who are assessed as ‘abnormal’ derive not from biology, but from implicit social judgments about the acceptability of certain kinds of biological variation”. Moreover, we should not be blind to the normative understandings of sexuality (i.e. heteronormativity) which is hidden behind these medical discourses on genital normality. An at first sight less problematic application of the term normal was its use as a statistical average in association with hormone levels. Still, in many cases, no reference range was provided.  And even if a reference range was provided, we should keep in mind that laboratory measurements of hormones are subject to variability and that reference ranges can vary from laboratory to laboratory (Lazarou et al., 2006; Symonds et al., 2020; Topliss, 2020). The most controversial use of normal was, without a doubt, in reference to quality of life, personhood and gender identity as in these cases normality was clearly used in an evaluative sense.

There did not seem to be a direct link between the use of normal/normality and the recommendation of early genital surgery insofar also studies who were in favor of deferring surgery frequently used references to normality. Still, as shown by Streuli and colleagues (Streuli et al., 2013), parental decisions with regard to medical treatment are profoundly influenced by the kind of information they receive from healthcare professionals. Talking about variations of sex characteristics in terms of (ab)normality can therefore implicitly incite a desire for normalization in both parents and patients and shape social attitudes. The study by Guntram (2013), for example, shows how women with VSC both align with and try to expand cultural norms about normal female embodiment by presenting themselves as differently normal” (i.e. as slightly different from the norm) or “normally different” (i.e. normalcy of variety).

Only a few studies in our sample were consciously aware of the impact of healthcare professionals’ language on patients’ self-perception, medical decisions and care (Dear et al., 2019; Hertweck & Rothstein, 2019; Roen et al., 2018) and expressed the need to pro-actively question the socio-cultural norms that underlie this word use.

Finally, only a minority of the studies included in the review come from the Middle-East, Latin-America and Africa, highlighting the geo-political overrepresentation of Anglo-phone and Western approaches to and understanding of intersex or DSD. The risk of imposing liberal notions such as the paradigm of patient-centred care on other care contexts has been emphasized by previous studies (David, 2015; Magubane, 2014) and was alluded to in some of the selected studies. That does not mean to say that people outside the western world would not benefit from individualized health care but that we should be wary of intersex imperialism (Kerry, 2011), “the imposition of Western conceptions of atypically sexed bodies and of how best to treat and respond to such bodies” (David, 2015, p. 74). Moreover, the paradigm of patient-centered care risks to focus mainly on patients and their bodies rather than on transforming the social world they inhabit.

## Limitations

Relevant studies might have gone unnoticed due to language bias and inclusion/exclusion criteria. Furthermore, only those articles that included normal/normality in the abstract or the main text were included in the review. Studies that used synonyms such as “common”, or “typical” were excluded. Those papers that did not use notions of normality might be more aware of the critical literature from the humanities and be more mindful of the fact that words can hurt. Our research results should thus be interpreted with caution. Still, our review offers an important insight in the use of normal/normality in (at least a part of) the medical literature on DSD and intersex. As such, it provides an opportunity for critical reflection among healthcare professionals who care for patients born with variations of sex characteristics.

## Conclusion

Normal and normality are frequently used in the medical literature on intersex or DSD. In order to provide better care to persons with VSC, healthcare professionals need to consider the potential dangerous implications of their word choice on person’s self-perception and medical decision-making and be mindful of the ethical implications of using photographs in publications given that many intersex persons describe their experience with medical photography as dehumanizing. Paying attention to patients’ lived experiences and making room for interdisciplinary research and publications in medical journals might be a promising way forward to wake up the sleeping socio-cultural metaphors of normality in medicine.
